# Cooperating, congenital neutropenia–associated *Csf3r* and *Runx1* mutations activate pro-inflammatory signaling and inhibit myeloid differentiation of mouse HSPCs

**DOI:** 10.1007/s00277-020-04194-0

**Published:** 2020-08-03

**Authors:** Malte Ritter, Maksim Klimiankou, Olga Klimenkova, Axel Schambach, Dirk Hoffmann, Amy Schmidt, Lothar Kanz, Daniel C. Link, Karl Welte, Julia Skokowa

**Affiliations:** 1 Division of Translational Oncology, Department of Hematology, Oncology, Clinical Immunology and Rheumatology, University Hospital Tübingen, Otfried-Müller-Straße 10, 72076 Tübingen, Germany; 2 Institute of Experimental Hematology, Hannover Medical School, Hannover, Germany; 3 Division of Hematology/Oncology, Boston Children’s Hospital, Harvard Medical School, Boston, MA USA; 4 Division of Oncology, Section of Stem Cell Biology, Washington University Medical School, St. Louis, MO USA; 5 The University Children’s Hospital Tübingen, Tübingen, Germany

**Keywords:** Severe congenital neutropenia, Pre-leukemia bone marrow failure syndrome, G-CSFR mutations, RUNX1 mutations

## Abstract

Patients with the pre-leukemia bone marrow failure syndrome called severe congenital neutropenia (CN) have an approximately 15% risk of developing acute myeloid leukemia (AML; called here CN/AML). Most CN/AML patients co-acquire *CSF3R* and *RUNX1* mutations, which play cooperative roles in the development of AML. To establish an in vitro model of leukemogenesis, we utilized bone marrow lin^−^ cells from transgenic C57BL/6-d715 *Csf3r* mice expressing a CN patient–mimicking truncated *CSF3R* mutation. We transduced these cells with vectors encoding *RUNX1* wild type (WT) or *RUNX1* mutant proteins carrying the R139G or R174L mutations. Cells transduced with these *RUNX1* mutants showed diminished in vitro myeloid differentiation and elevated replating capacity, compared with those expressing WT *RUNX1*. mRNA expression analysis showed that cells transduced with the *RUNX1* mutants exhibited hyperactivation of inflammatory signaling and innate immunity pathways, including IL-6, TLR, NF-kappaB, IFN, and TREM1 signaling. These data suggest that the expression of mutated *RUNX1* in a *CSF3R*-mutated background may activate the pro-inflammatory cell state and inhibit myeloid differentiation.

**Electronic supplementary material:**

The online version of this article (10.1007/s00277-020-04194-0) contains supplementary material, which is available to authorized users.

## Introduction

Patients with the inborn pre-leukemia bone marrow failure syndrome called severe congenital neutropenia (CN) have a very low level (less than 500 cells/μl blood) or even a complete lack of mature neutrophilic granulocytes in the peripheral blood which is caused by blockade of the terminal differentiation of bone marrow myeloid progenitor cells at the promyelocytes/myelocyte stage [1, 2]. In most CN patients, this granulocyte differentiation defect can be successfully treated by daily subcutaneous administration of recombinant human granulocyte colony–stimulating factor (G-CSF). Approximately 15% of CN patients develop myelodysplastic syndrome or acute myeloid leukemia (MDS or AML). Inherited mutations in the ELANE, HAX1, G6PC3, SRP54, GFI1, and JAGN1 genes cause CN, and leukemic progression has been seen in CN patients of all genetic groups [1].

*CSF3R* mutations resulting in the production of truncated G-CSFR proteins that lack from one to four phospho-tyrosine residues and exhibit defective receptor internalization were reported in a majority of CN patients with overt AML or MDS [3–9]. However, transgenic d715 *Csf3r* mice lacking three tyrosines do not develop AML or MDS [3–9], suggesting that additional genetic alterations in combination with *CSF3R* mutation are needed for the progression of AML. We recently examined a large cohort of CN/AML patients (31 patients) and found cooperative acquired mutations of *CSF3R* and *RUNX1* (runt-related transcription factor 1) in 55% of CN patients with overt AML or MDS [10]. However, the detailed mechanism underlying the leukemogenic transformation downstream of *CSF3R* and *RUNX1* mutations remained unknown.

Acquired mutations in *RUNX1* occur in AML, mostly secondary to MDS, radiation therapy, or chemotherapy [11–16]. Most *RUNX1* mutations are acquired heterozygous point mutations; they are predominantly located in the Runt homology/DNA-binding (RHD) or transactivation (TAD) domains. Interestingly, a majority of patients with familial platelet disorder (FPD) and a predisposition for AML have germline *RUNX1* mutations [17]. Some FPD patients with overt AML gain additional *RUNX1* mutations [17]. Among the described groups of AML patients, the incidence of acquired *RUNX1* mutations is the highest in CN/AML patients. *RUNX1* mutations in CN/AML patients are distributed throughout the RHD (primarily) and TAD of the RUNX1 protein, and some hot spot positions have been noted [10]. For example, amino acid residues 139 and 174 of the RUNX1 protein were found to be mutated in four and three CN/AML patients, respectively [10] (data not shown). The functional outcomes of *RUNX1* mutations at different positions have not yet been clearly defined, but we speculate that they may affect the DNA binding of RUNX1 to target genes or the protein–protein interactions, intracellular localization, protein stability, and/or post-translational modification(s) of RUNX1.

The role of inflammation in cancer was first mentioned in 1863 by Virchow [18]. A growing body of research suggests that pro-inflammatory signaling acts through diverse mechanisms to increase the proliferation rate of hematopoietic stem and progenitor cells (HSPCs), which induces genotoxicity, increases survival, and produces pre-leukemia stem cells (pre-LSCs) with a high likelihood of leukemic transformation [19].

In the present study, we sought to establish an in vitro experimental model to study the intracellular mechanisms of leukemia development downstream of *CSF3R* and *RUNX1* mutations. Using this model, we identified upregulation of an inflammatory signature signaling in mouse HSPCs expressing mutated *CSF3R* and *RUNX1*. This expression signature may predispose CN patients toward leukemic transformation.

## Material and methods

### Mice

Male d715 *Csf3r* mice on the C57BL/6J background have been described previously [9]. Mice were housed under pathogen-free conditions in the animal facility of Tübingen University.

### Cell purification and separation

Mouse bone marrow cells were isolated by flushing the long bones with ice-cold PBS. Bone marrow mononuclear cells were isolated by Ficoll–Hypaque gradient centrifugation (Amersham Biosciences) and positively selected bone marrow lin^−^ cells by immunomagnetic labeling with corresponding MACS beads (Miltenyi Biotec). Cells were counted, and viability was assessed by Trypan blue dye exclusion.

### Generation of the lentiviral vectors expressing WT or mutant RUNX1 cDNA

To generate RUNX1 mutants, we performed site-directed mutagenesis. As a template, we used Lego-iG/Puro-RUNX1-wt-CTAP plasmid expressing human wild type (WT) RUNX1 generously provided by Dr. Boris Fehse and Dr. Carol Stocking. Specific primers to introduce mutations p.Arg139Gly (R139G) and p.Arg174Leu (R174L) in wild type *RUNX1* nucleotide sequence were designed using the QuickChange Primer Design tool (https://www.agilent.com/store/primerDesignProgram.jsp). Primer sequences are available upon request. Lego-iG/Puro-RUNX1-R139G-CTAP and Lego-iG/Puro-RUNX1-R174L-CTAP plasmids were generated using the QuickChange II Site-Directed Mutagenesis Kit (Agilent Technologies, Inc.) according to the manufacturer’s instruction. *RUNX1* cDNAs (WT and two mutants) were subsequently re-cloned into lentiviral pRRL.PPT.CBX3.SFFV.hRUNX1.i2.EBFP.puro.pre vector. Transgene expression was controlled by the spleen focus–forming virus promoter (SFFV) juxtaposed to the minimal ubiquitous chromatin opening element (CBX3). EBFP translation was initiated by an internal ribosomal entry side (i2).

### Transduction of cells

Lin^−^ cells were cultured in a hematopoietic stem cell expansion medium consisting of Stemline II medium supplemented with Pen/Strep, 10% FCS (Sigma-Aldrich), 1 μm dexamethasone, 100 ng/ml of mSCF, 4 ng/ml of mIL-3, 10 ng/ml of hIL-6, 40 ng/ml of murine IGF-1, and 20 ng/ml of human Flt-3L at 2 × 10^5^ cells/ml for 2 days. Cells were transduced at MOIs of 5–10 in the presence of polybrene (5 μg/ml) and re-transduced after 12–24 h. Percentage of EBFP^+^ cells was assessed by FACS.

### Detection of the human RUNX1 protein in transduced bone marrow lin^−^ cells from d715 *Csf3r* mice using western blotting

A total of 1 × 10^6^ transduced bone marrow lin^−^ cells from d715 Csf3r mice were lysed in 200 μl 3 × Laemmli buffer, and protein was denatured for 10 min at 95 °C. Five microliter of cell lysate in Laemmli buffer was loaded per lane. Proteins were separated on a 12.5% polyacrylamide gel and transferred on a nitrocellulose membrane (GE Healthcare) (1 h, 100 V, 4 °C). The membrane was blocked for 1 h in 5% milk and incubated with primary rabbit anti-human and anti-mouse RUNX1-specific (Cell Signaling Technology #4334) or GAPDH (Cell Signaling Technology, #2118) antibody overnight (at 4 °C). After that, membranes were washed and incubated with secondary HRP-conjugated antibody (Cell signaling, #7074) for 1 h at room temperature. Pierce ECL solution (Thermo Fisher) and Amersham Hyperfilms were used to detect chemiluminescence signal of proteins.

### Liquid culture differentiation of transduced lin^−^ cells

A total of 2 × 10^5^ of transduced cells/ml were incubated for 7 days in RPMI 1640 GlutaMAX supplemented with 10% FCS, 10 ng/ml hIL-6, 5 ng/ml IL-3, 5 ng/ml GM-CSF, and 10 ng/ml G-CSF. The medium was exchanged every second day. On day 7, the medium was changed to RPMI 1640 GlutaMAX supplemented with 10% FBS, 1% penicillin/streptomycin, and 10 ng/ml G-CSF. The medium was exchanged every second day until day 11. On day 11, cells were analyzed by FACS using the following antibody: rat anti-mouse Gr-1 (BD 553128) and rat anti-mouse CD11b (BD 553312) on FACSCanto II.

### Colony-forming unit assay

A total of 1000 transduced EBFP^+^ cells were plated directly after transduction in 1 ml methylcellulose medium (MethoCult GF M3434; StemCell Technologies) supplemented with 10 ng/ml of G-CSF. After 14 days of culture, the numbers of CFU-G, CFU-GM, and BFU-E colonies were counted. Cells were collected for the colony replating experiments, washed 3 times with PBS, and plated 1000 cells/dish in new methylcellulose for an additional 2 weeks (1st replating). The procedure was repeated one more time (2nd replating).

### Microarray-based mRNA expression analysis

After 48 h of lentiviral transduction, lin^−^ cells were starved for 24 h and subsequently treated with 10 ng/ml of G-CSF for 24 h. After that, transduced cells were sorted, and mRNA was isolated using the RNeasy Mini Kit (Qiagen, #74106) according to the manufacturer’s instructions.

RNA from transduced lin^−^ cells was subjected to microarray analysis using the Affymetrix Microarray Platform. The GeneChip WT cDNA Synthesis and Amplification Kit was used to make double-stranded cDNA from total RNA, which was then labeled with biotin (GeneChip WT Terminal Labeling Kit). After chemical fragmentation of the biotin-labeled cDNA targets, they were hybridized to the GeneChip Mouse Gene 2.0 ST Array using the Fluidics Station 450 and scanned using the Affymetrix GeneChip Scanner 3000 with the GeneChip Operating Software 1.4 (Affymetrix, Santa Clara, CA). Data analysis was performed using Affymetrix Expression Console Version 1.1 for invariant set normalization, and the Ingenuity Pathway Analysis (IPA) software (Qiagen) was used for identification of differentially expressed genes. Motif activity response analysis (MARA) was conducted using the Integrated System for Motif Activity Response Analysis (ISMARA); CEL files were uploaded to the server using the web interface.

### Cytospin preparation, staining, and microscopic image acquisition

After sorting, 1 × 10^4^ cells were centrifuge onto microscope slides at 250 rpm for 3 min. The slides were air-dried, and subsequent Wright–Giemsa staining was carried out. Images where acquired on a Nikon Eclipse TS100. Cells were covered with oil, and images were collected at × 630 magnification.

### qRT-PCR

RNA isolation was performed using the RNeasy Micro Kit (Qiagen). cDNA was synthesized from 1 μg total RNA with the Omniscript RT Kit (Qiagen). qRT-PCR was conducted with SYBR Green qPCR master mix (Roche) on a Light Cycler 480 (Roche). Target genes were normalized to ACTB. Primers are available upon request.

### LSK cell analysis

A total of 2 × 10^5^ cells were incubated with FcR blocking antibody (BioLegend #101320), 7AAD, and lineage cocktail biotin–conjugated antibody (BioLegend #79750, #79748, #79752, #79748, #79749), and stained with APC-Cy7-conjugated streptavidin (BioLegend #405208), anti-mouse Sca-1 BV510–conjugated antibody (BioLegend, #108129), and anti-mouse-c-KIT APC–conjugated antibody (BioLegend, #105812). After staining and washing, cells were analyzed on a CANTO II (BD) flow cytometer.

### Statistics

Statistical analysis was performed using a two-sided unpaired Student *t* test for the analysis of differences in mean values between groups.

## Results

### Diminished formation of myeloid colonies and elevated replating capacity of d715 *Csf3r* lin^−^ cells transduced with mutated *RUNX1*

**Fig. 1:**
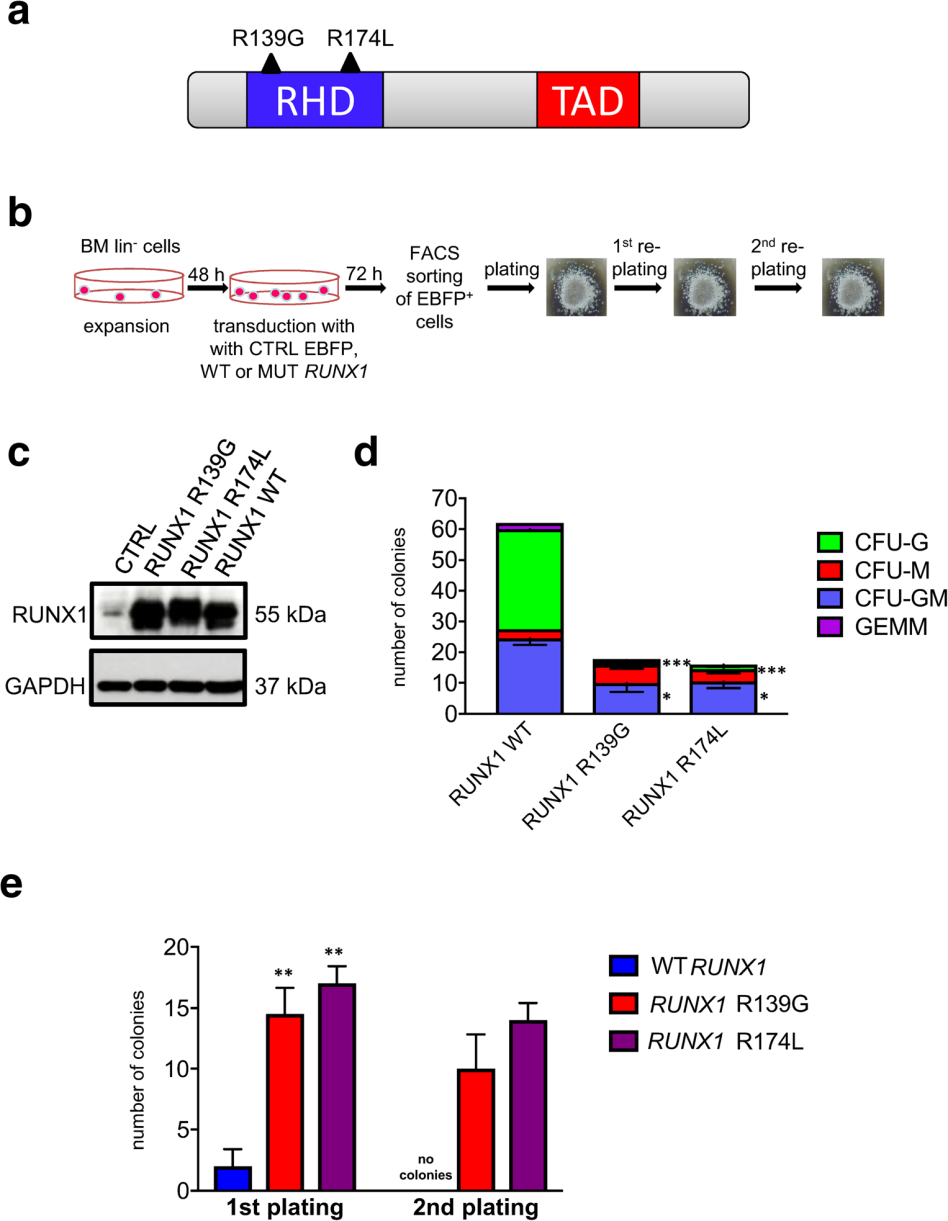
In HSPCs of d715 *Csf3r* mice, *RUNX1* mutations decrease CFU-G and CFU-GM formation but increase the replating capacity. **a** Schematic of RUNX1 protein showing the location and amino acid changes of the mutations, which are indicated by black triangles. The functionally important Runt homology DNA-binding domain (RHD) is shown in blue, and the transactivation domain (TAD) is shown in red. **b** Schematic of CFU experiments performed using transduced bone marrow lin^−^ cells of C57BL/6-d715 *Csf3r* mice. **c** Representative WB images of lin^−^ cells of C57BL/6-d715 *Csf3r* mice transduced with corresponding lentiviral constructs. GAPDH was used as loading control. **d** CFU assay and **e** replating CFU assay of transduced C57BL/6-d715 *Csf3r* lin^−^ cells. Data represent means ± SD from triplicates of two independent experiments; *p < 0.05, **p < 0.01,*** *p* < 0.001

We isolated bone marrow lin^−^ cells from d715 *Csf3r* mice and transduced these cells with lentiviral vectors encoding EBFP and *RUNX1* wild type (*RUNX1*-WT), or mutants, *RUNX1*-R139G, or *RUNX1*-R174L. Amino acids 174 and 139 were found to be “hot spots” for mutation in the RHD domain of the RUNX1 protein, as they were detected in four and three CN-AML patients, respectively (Fig. 1a) [10] (data not shown). At 72 h post-transduction, we performed colony-forming units and replating experiments of sorted EBFP^+^ cells (Fig. 1b). We observed comparable expression of human WT and mutant RUNX1 proteins in transduced cells (Fig. 1c). Low levels of endogenous murine runx1 protein were detected in BFP^+^ control transduced cells (Fig. 1c). Interestingly, d715 *Csf3r* lin^−^ cells transduced with mutated *RUNX1* had markedly diminished capacities to form myeloid colonies, including CFU-G and CFU-GM, as compared with cells transduced with WT *RUNX1* (Fig. 1d). In line with these findings, CFU replating experiments showed that cell transduced with each *RUNX1* mutant had markedly higher replating capacities than *RUNX1* WT–transduced cells (Fig. 1e).

### Reduced liquid culture myeloid differentiation of d715 *Csf3r* lin^−^ cells transduced with mutated *RUNX1*

**Fig. 2:**
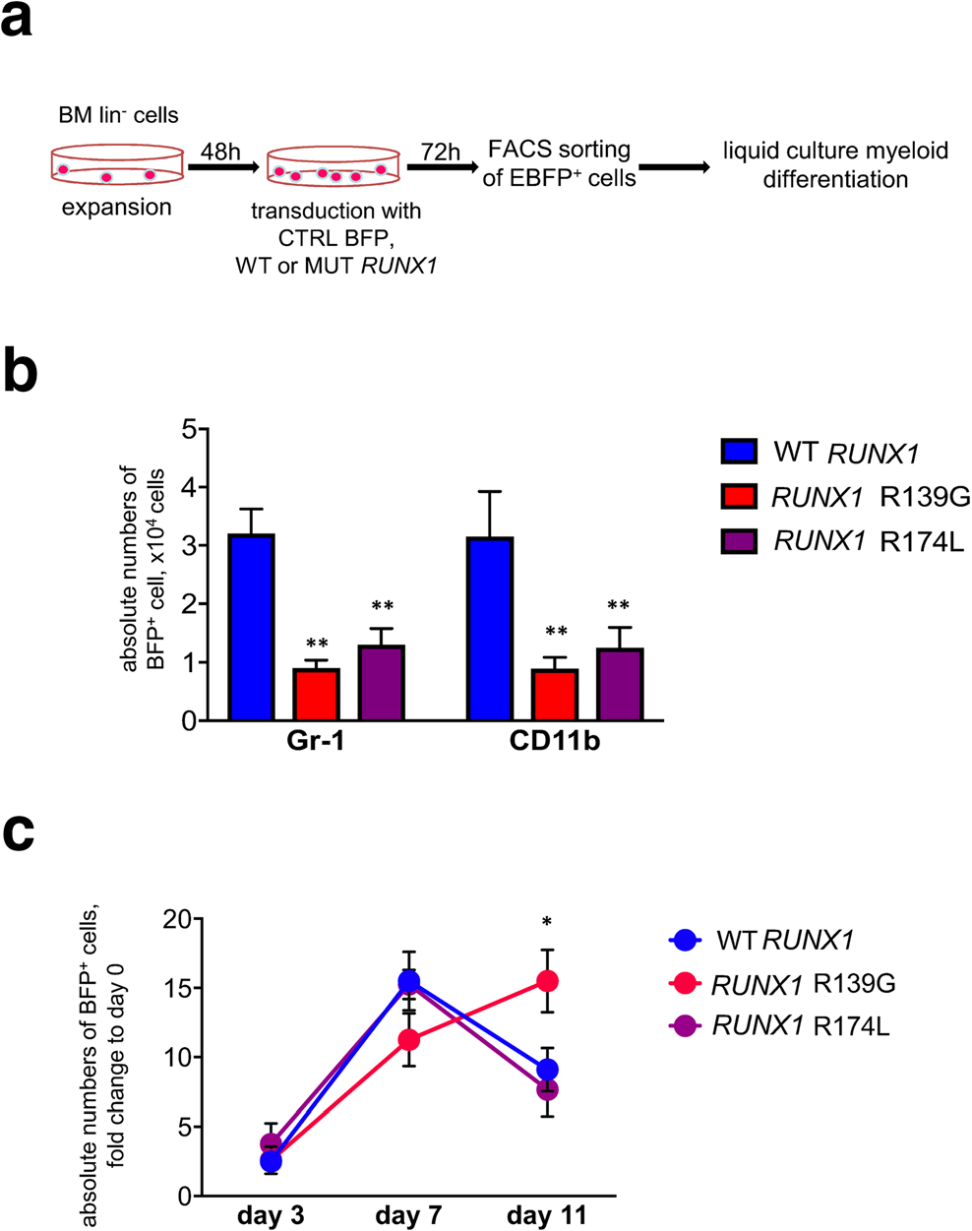
Liquid culture differentiation of transduced d715 *Csf3r* HSPCs. **a** Schematic of the workflow for liquid culture myeloid differentiation of transduced bone marrow lin^−^ cells obtained from C57BL/6-d715 *Csf3r* mice. **b** Transduced cells were subjected to liquid culture myeloid differentiation (see “Material and methods” for details). FACS was used to count the myeloid and granulocytic cells on day 11 of culture. Graph bars represent absolute cell counts of Gr-1^+^ or CD11b^+^ cells. Data represent means ± SD from triplicates of two independent experiments; **p < 0.01

We next compared the G-CSF-triggered myeloid differentiation of transduced d715 *Csf3r* lin^−^ cells in vitro. Transduced cells were cultured in liquid culture myeloid differentiation medium for 11 days (Fig. 2a). FACS was used to count the absolute numbers of myeloid CD11b^+^ and Gr-1^+^ cells on day 11 of liquid culture, and the results showed that d715 *Csf3r* lin^−^ cells transduced with each of the *RUNX1* mutants exhibited reduced myeloid differentiation, compared with WT *RUNX1*–overexpressing samples (Fig. 2b). At the same time, no marked difference was observed in the absolute numbers of BFP^+^ cells between groups transduced with WT *RUNX1* or with each of *RUNX1* mutants on days 3 and day 7 of liquid culture differentiation. On day 11 of culture, numbers of BFP^+^ cells transduced with *RUNX1*-R139G mutant were significantly (*p* < 0.05) increased, as compared with *RUNX1* WT– or *RUNX1*-R174L–transduced samples (Fig. 2c).

### *RUNX1* missense mutations induce inflammatory signaling pathways in G-CSF-treated d715 *Csf3r* hematopoietic cells

**Fig. 3:**
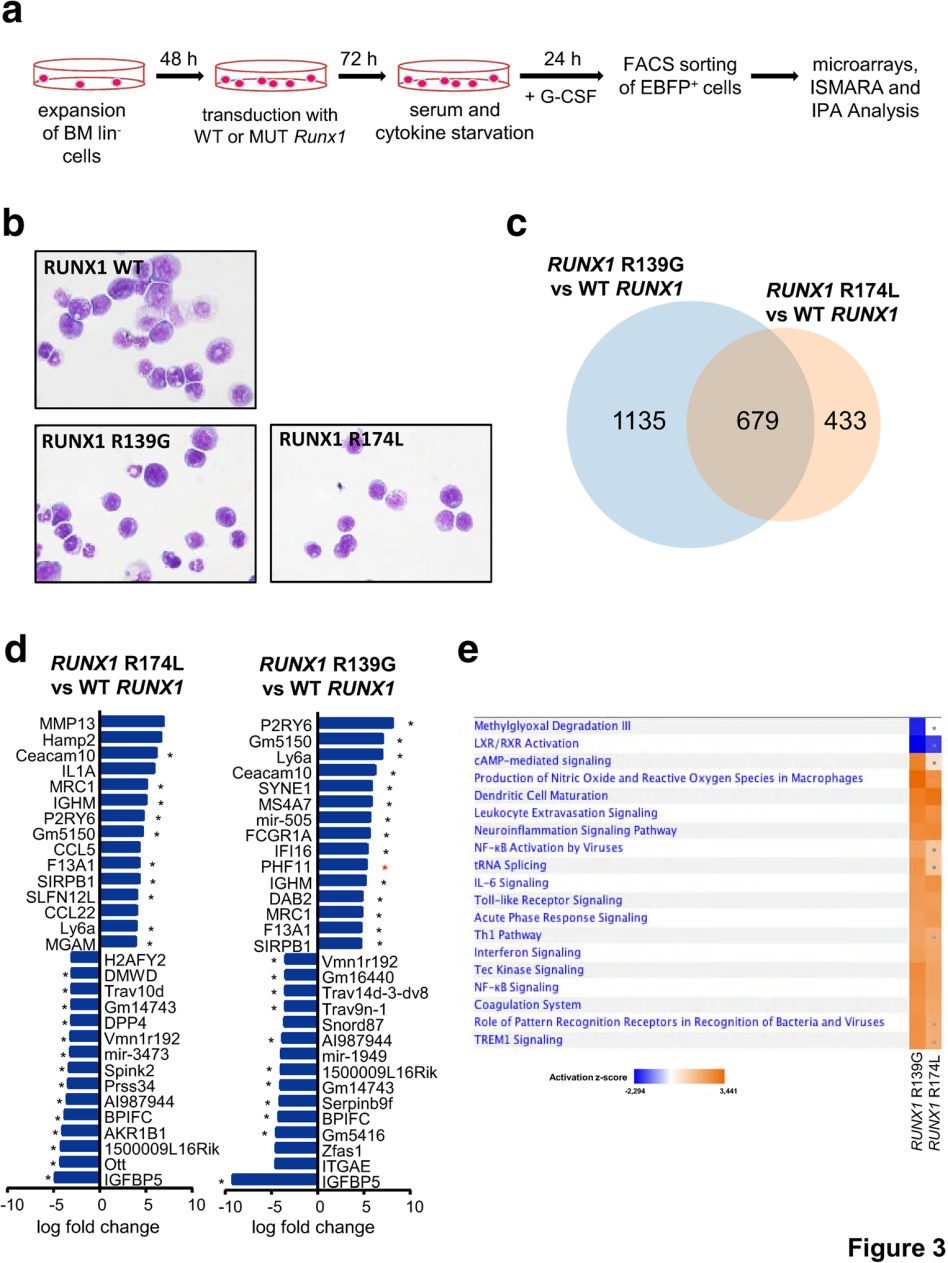
Canonical pathway analysis of microarray data obtained from transduced d715 *Csf3r* HSPCs. **a** Schematic of the experimental procedure performed for microarray analysis. The experiment was conducted in duplicate. After quality control analysis, one sample from the *RUNX1*-R139G group was excluded from the final analysis. **b** Wright–Giemsa–stained cytospin preparations of *Csf3r* lin^−^ cells transduced with *RUNX1* WT and mutants and sorted for fluorescent protein expression. Images were acquired at × 630 magnification. **c** Venn diagram depicting the overlay of significantly up- or downregulated transcripts in each *RUNX1* mutant group, as compared with WT *RUNX1*–transduced cells. **d** Canonical pathways that were significantly (−log(*p* value) > 1.3) enriched and significantly predicted (*z*-value > 2 and < − 2) to be up- or downregulated in each *RUNX1* mutant group compared with WT *RUNX1*–transduced cells. Shared pathways are marked with an asterisk (*). **e** IPA analysis of the significantly regulated pathways shared by lin^−^ cells transduced with each *RUNX1* mutant, as compared with WT *RUNX1*–overexpressing samples. Upregulated pathways are shown in orange, and downregulated pathways are shown in blue

We further aimed to identify intracellular signaling pathways downstream of *csf3r* and *RUNX1* mutations that may affect the in vitro proliferation and myeloid differentiation of mouse HSPCs. The d715 *Csf3r* lin^−^ cells were expanded in HSPC expansion medium and transduced with lentiviral vectors carrying *RUNX1* WT, *RUNX1*-R139G, or *RUNX1*-R174L. Transduced cells were starved for 24 h and then treated with G-CSF for 24 h. EBFP^+^ cells were sorted in RLT buffer, and mRNA expression was evaluated using an Affymetrix MoGene 2.0 Chip (Fig. 3a). Representative images of Wright–Giemsa–stained cytospins prepared from sorted cells show no difference in the cell morphology between studied groups: all samples show immature cell morphology (Fig. 3b). These data suggest that the differences in mRNA expression should not contribute to a strong diversity in the cell composition between studied groups.

After invariant set normalization, the expression levels of the *RUNX1* mutants were normalized to those obtained from WT *RUNX1*–transduced cells. The fold change expression table (Suppl. Table 1) was uploaded to the IPA software, and the cutoff values were set to − 1.7 and + 1.7 for log fold change, with the goal of identifying differentially expressed genes. This analysis showed that 1113 and 1814 genes were differentially expressed in the *RUNX1*-R174L and *RUNX1*-R139G mutant groups compared with the WT *RUNX1* group (Suppl. Table 2). Overlap analysis revealed that 679 genes (37.4% for the *RUNX1*-R139G mutant and 61% for the *RUNX1*-R174L mutant) were co-regulated in both *RUNX1* mutant groups (Fig. 3c). The 15 most highly up- and downregulated genes were very similar between the *RUNX1* mutant groups. Of the top upregulated genes, 10/15 from the *RUNX1*-R174L group and 14/15 from the *RUNX1*-R139G group were co-activated in both groups. Of the top downregulated genes, 14/15 from the *RUNX1*-R174L group and 11/15 from the *RUNX1*-R139G group were co-downregulated (Fig. 3d). A comparison analysis performed using the IPA software revealed that 46 and 79 canonical pathways were significantly enriched (log(*p* value) ≥ 1.3 corresponding to *p* ≤ 0.05) in d715 *Csf3r* lin^−^ cells transduced with *RUNX1*-R174L or *RUNX1*-R139G, respectively. Of these pathways, 11 in the *RUNX1*-R174L group and 18 in the *RUNX1*-R139G group were significantly activated (*z*-value ≥ 2) or inhibited (*z*-value ≤ − 2). Most of the regulated pathways belonged to members of the activated innate immune pathways category; these included NF-kappaB signaling, toll-like receptor signaling (TLR), acute-phase response signaling, production of nitric oxide and reactive oxygen species, pattern-recognizing receptors, Trem 1 signaling, and IL-1 signaling. Activation was also seen among members of the inflammatory signaling pathways, such as IL-6 signaling, interferon signaling, Tec kinase signaling, and leukocyte extravasation signaling (Fig. 3e, Suppl. Table 3).

### Representative pathways of the innate immune system activated in d715 *Csf3r* lin^−^ cells transduced with *RUNX1* mutants

As examples of the activated canonical inflammatory and innate immune signaling pathways, canonical TLR signaling and IL-6 pathways are depicted in Suppl. Figs. 2 and 3. TLR signaling was upregulated in d715 *Csf3r* lin^−^ cells transduced with the R139G or R174L *RUNX1* mutants; this resulted in activation of transcription factors through Janus kinase 1 (JAK1) and p38 MAPK, promoting the expression of IL-1, TNF-alpha, and IL-12 (Suppl. Fig. 2A, B). Interferon signaling acted through JAK1 and JAK2 to activate the transcription factors, STAT1 and STAT2, thereby upregulating interferon response factors and other pro-inflammatory genes (Suppl. Fig. 2A, B).

The upregulation of IL-6 signaling–dependent genes in d715 *Csf3r* lin^−^ cells transduced with each *RUNX1* mutant was also mediated through JAK signaling, but relied on the transcription factor, STAT3, to confer translational regulation in the nucleus. Additional IL-6-dependent activation of ERK1 induced the NF-kappaB-NF-IL-6 transcription factor complex (Suppl. Fig. 3A, B).

### Upstream pathway analysis points toward inflammatory dysregulation

Upstream analysis performed using the IPA software identified potential upstream regulators responsible for the gene expression signatures observed in the studied groups. We detected 768 upstream regulators in the *RUNX1*-R139G mutant group and 875 regulators in the *RUNX1*-R174L mutant group (Suppl. Fig. 4A, Suppl. Table 4). Five hundred upstream regulators were commonly found in both groups: 65.1% in the *RUNX1*-R139G mutant group and 57.1% in the *RUNX1*-R174L mutant group (Suppl. Fig. 4A). Among the 15 top activated upstream regulators, 9/15 for *RUNX1*-R174L and 11/15 for *RUNX1*-R139G were shared between the groups. Among the 15 top downregulated regulators, overlaps were seen in 6/15 for *RUNX1*-R174L and 9/15 for *RUNX1*-R139G (Suppl. Fig. 4B). Selected up- and downregulated candidate genes were validated by qRT-PCR in the independent set of experiments (Suppl. Fig. 4C). Also, staining of transduced G-CSF-treated lin^−^ cells with Sca-1 and c-kit antibody followed by flow cytometry analysis revealed an increase of lin^−^Sca-1^+^c-KIT^+^ cell population in *RUNX1*-R139G– and *RUNX1*-R174L–transduced groups, compared with cells expressing *RUNX1* WT, or GFP CTRL (Suppl. Fig. 4D).

**Fig. 4:**
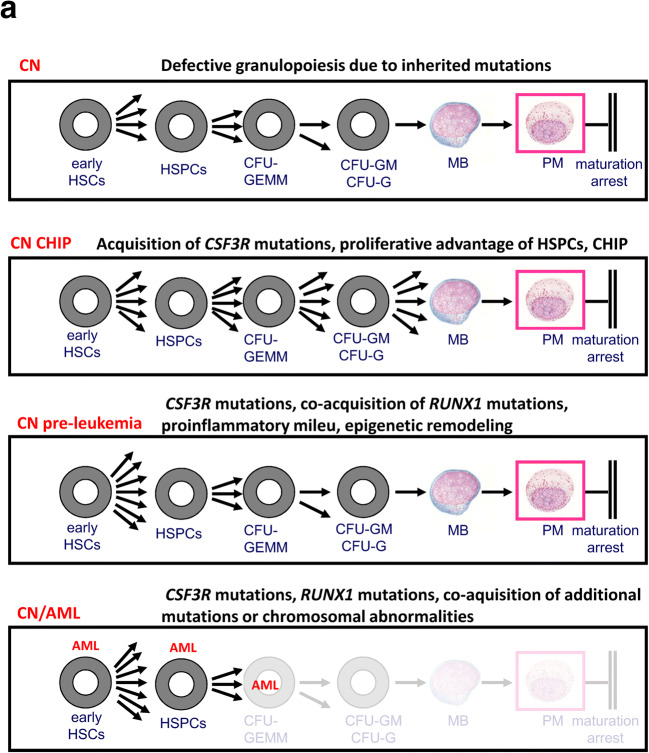
Proposed model for leukemia development in CN. **a** CN, CN-associated germline mutations cause maturation arrest of granulopoiesis at the stage of promyelocytes/myelocyte. CN CHIP, HSPCs that acquire *CSF3R* mutations gain a proliferative advantage that may mimic the CHIP phenotype. CN pre-leukemia, the co-acquisition of *RUNX1* mutation induces an inflammatory milieu, leading to genotoxicity, additional defects of myeloid differentiation, and elevated proliferation that constitute the pre-leukemia stage. CN/AML, the acquisition of additional leukemia-associated gene mutations or chromosomal abnormalities results in AML or MDS

The top overlapping upstream regulators included a number of inflammatory cytokines, such as IL-15 and IL-18. IL-15 has been described as a responsible driver for chronic inflammation in autoimmune diseases and hematological malignancies [20]. Both IL-15 and IL-18 are confirmed targets for antitumor activity [21, 22]. Another factor that promotes survival of leukemogenic cells, Bcl2a1 [23], was also highly expressed, as were Ly6a and Sca-1, which are known as stemness factors for HSPCs.^24^ The top downregulated genes included anti-inflammatory genes, such as Il-10 [25], and the cytokine-processing factor, Dpp4, which can increase proliferation by prolonging cytokine signaling [26]. Interestingly, *Csf3r* was among the highest expressed genes in both groups. This is in accordance with the literature showing that G-CSF sensitivity is increased in AML blasts with missense *RUNX1* mutations.

### Motif activity response analysis confirms the central role of inflammatory and innate immunity signaling and the presence of early molecular changes related to MDS/AML downstream of Csf3r and *RUNX1* mutations

We used the ISMARA [27] web tool to analyze the transcription factor binding motifs enriched among the differentially expressed genes. We found that the most highly enriched motif was Irf2_Irf1_Irf8_Irf9_Irf7 (*z*-value 4.564), which corresponds to the interferon regulatory factors. This indicates that an interferon-related change in inflammation signaling is responsible for at least some of the observed gene expression differences. The Stat2 motif was significantly correlated with the expression signature (*z*-value 2.707), and we observed significant activation of the Klf4_Sp3 (*z*-value 2.349) and Mecp-2 (*z*-value 2.627) transcription factor binding motifs known to be associated with AML or MDS, but significant downregulation of the Max–Mycn motif (*z*-value 2.016), upon transduction of d715 *Csf3r* lin^−^ cells with *RUNX1* mutants (Suppl. Fig. 5A). Finally, our inferred activity analysis of each motif revealed that there was a high degree of similarity in the significantly activated motifs associated with the two different *RUNX1* mutants (Suppl. Fig. 5B).

## Discussion

The identification of cooperative *CSF3R* and *RUNX1* mutations in a majority of CN patients with overt MDS or AML brought us one step closer to understanding leukemia development [3]. Truncated *CSF3R* mutations are a very rare event in de novo AML, while *RUNX1* mutations are most frequent among patients with secondary AML (sAML) after chemotherapy or radiation therapy, which represents about 30% of adult AML cases. Acquired cooperative *CSF3R* and *RUNX1* mutations in approximately 55% of CN/AML patients are a unique feature and may reflect the inherited background of patients with CN-associated gene mutations, such as those in *ELANE* or *HAX1*. Mechanistically, *ELANE* mutations induce unfolded protein response (UPR) [28, 29] and endoplasmic reticulum (ER) stress, while mutations in *HAX1* have pro-apoptotic functions [30, 31]. The role of inherited CN-associated mutations in the process of leukemogenesis needs to be investigated. The presence of these mutations may predispose to acquisition of secondary mutations in *CSF3R* and *RUNX1* and the development of leukemia. Similar to secondary leukemias, wherein HSPCs are damaged by chemotherapeutic agents or radiation therapy, inherited CN-specific mutations may induce DNA damage or stress responses in HSPCs. Indeed, using CN patient–derived iPSCs as an experimental model, we recently showed that an elevation of DNA damage in CN HSPCs precedes the leukemic transformation [32]. In the present study, we found that *csf3r* and *RUNX1* mutations in HSPCs induce reduced myeloid differentiation and enhanced clonogenic potential. Our findings demonstrated that cells transduced with WT *RUNX1* differentiated and lost the capacity to proliferate, making no colonies in replating experiments. In contrast, cells transduced with *RUNX1* mutants differentiate less, but retain proliferative potential. Leukemogenic activity of *csf3R* and *RUNX1* mutations should be further elaborated in vivo using a mouse model.

Elucidation of the deregulated intracellular signaling pathways downstream of *CSF3R* and *RUNX1* mutations will help to identify druggable targets, with the goal of eliminating leukemia-predisposed HSPCs and/or specifically targeting CN/AML blasts. Interestingly, mRNA expression analysis revealed that HSPCs harboring mutations in *Csf3R* and *RUNX1* exhibit activation of the inflammatory and innate immunity pathways, including interferon signaling; IL-1, IL-6, IL-8, and TLR signaling; and TREM1 signaling. Additionally, we also found marked upregulation of Ly6a (also known as Sca-1), a marker of stemness in HSPCs [24] that is also upregulated upon inflammation [33–35]. IL-6 and IL-8 were previously known to be hyper-activated in MDS and AML [19, 36, 37]. Moreover, the increased genotoxic stress in HSPCs of 40% de novo MDS cases has been associated with elevated TLRs/Myd88-triggered intracellular signaling and IL-8 expression [19]. Most probably, the presence of truncated *CSF3R* and *RUNX1* mutations in HSPCs of CN patients alters the pro-inflammatory cell state, enhances proliferation, and increases the susceptibility of HSPCs to genomic toxicity. Recent reports revealed that pre-leukemic HSPCs carrying an *ETV6*-*RUNX1* fusion gene or having *Pax5* haploinsufficiency evolved to precursor B cell acute lymphocytic leukemia upon activation of the pro-inflammatory pathways [38, 39]. The role of activated innate immunity and inflammatory pathways in the leukemogenesis of CN HSPCs was not studied yet. It would be interesting to investigate the susceptibility and expression kinetics of TLRs and the receptors for IL-1 and IL-8 on HSPCs from CN/AML patients during the development of leukemia.

We found that the Spi1/PU.1 transcription factor motif is activated in HSPCs downstream of *Csf3r* and *RUNX1* mutations. PU.1 is upregulated in hematopoietic cells of CN patients [40] and is an essential transcription factor for monocytic differentiation [41, 42]. PU.1 also acts as a maintenance factor for pre- and leukemia-initiating cells [43]. The leukemogenic function of upregulated PU.1 in HSPCs of CN patients has not been studied yet.

Taken together, our results support the following proposed mechanism for leukemia development in CN: *CSF3R* mutations represent a state of clonal hematopoiesis of indeterminate potential (CHIP) in CN patients in that they confer a clonal advantage. Co-acquisition of *RUNX1* mutation further increases proliferation and genotoxicity, including hypersensitivity to pro-inflammatory signaling. This combination may lead to acquisition of additional somatic mutations (e.g., in *SUZ12* or *ASXL1*) or chromosomal abnormalities (e.g., monosomy 7 or trisomy 21), which finally overt to MDS or AML (Fig. 4).

## Electronic supplementary material

Supplementary Figure 1 Selected significantly enriched canonical pathways detected by IPA core analysis. (PNG 610 kb)

High resolution image (TIF 1568 kb)

Supplementary Figure 2 **TLR and IFN pathways are hyper-activated in d715** ***Csf3r*** **HSPCs transduced with** ***RUNX1*** **mutants, as compared to WT** ***RUNX1*** **transduced cells.** Pathway analysis of differentially expressed genes between WT *RUNX1-*overexpressing d715 *Csf3r* lin^-^ cells and *RUNX1*-R139G- (A) or *RUNX1*-R174L (B) transduced cells was conducted using IPA software. Selected canonical signaling pathways (left: TLR signaling, right: Interferon signaling) that are activated in the presence of mutated *RUNX1*, in comparison to WT *RUNX1* are depicted. The overlay of all matching molecules of selected pathways in the IPA dataset is shown. Genes marked in red are upregulated in *RUNX1*-mutant groups, as compared to WT-*RUNX1* sampels, while green coloured genes are downregulated. (PNG 3476 kb)

High resolution image (TIF 7868 kb)

Supplementary Figure 3 **IL-6 signaling is upregulated in d715** ***Csf3r*** **HSPCs transduced with** ***RUNX1*** **mutants.** Overlays for IL-6 signaling pathway of all matching molecules contained in the IPA dataset demonstrate pathway activation in **A,** *RUNX1-*R139G and **B,** *RUNX1*-R174L overexpressing d715 *Csf3r* lin- cells compared to WT *RUNX1* overexpressing cells. Green represents negative expression fold change while red marks positive expression fold change. Genes marked in red are upregulated in *RUNX1*-mutant groups, as compared to WT-*RUNX1* sampels, while green coloured genes are downregulated. (PNG 2433 kb)

High resolution image (TIF 6083 kb)

Supplementary Figure 4 **Analysis of the upstream regulators responsible for the described phenotypes. A,** Venn diagram depicting the overlay of all significantly enriched upstream regulators. **B,** Among all significantly enriched upstream regulators found by IPA core analysis, the 15 top up- and downregulated upstream regulators are shown. Black stars mark genes that are differentially expressed in the same direction in both *RUNX1* mutants; red stars mark genes that are expressed oppositely. **C,** qRT-PCR of the selected candidate genes that were identified in the microarray analysis. The fold change of expression differences of cells transduced with *RUNX1* mutants normalized to *RUNX1* wildtype transduced samples is shown. **D,** Flow cytometry analysis of LSK cells in d715 *Csf3r* lin^-^ cell population transduced with *RUNX1*-WT, or *RUNX1* mutants and treated with G-CSF, as described in the Material and Methods section. Representative results of one donor mouse are shown. (PNG 599 kb)

High resolution image (TIF 1676 kb)

Supplementary Figure 5 **Motif Activity Response Analysis performed using the ISMARA webtool for d715** ***Csf3r*** **lin**^**-**^ **cells transduced with WT** ***RUNX1*** **or the** ***RUNX1*** **mutants A,** Significant active motifs found to be responsible for the observed differential gene expression patterns in d715 *Csf3r* lin^-^ cells transduced with *RUNX1* mutants compared to WT *RUNX1*-overexpressing cells (z-value >2). **B,** Activity scores (arbitrary unit) for each motif in the variously transduced cells. (PNG 253 kb)

High resolution image (TIF 1316 kb)

Supplementary Table 1 Gene lists used for IPA Ingenuity. (XLSX 2914 kb)

Supplementary Table 2 List of genes used in core analysis by IPA comparing expression profiles of *RUNX1* mutant transduced cells normalized to *RUNX1* WT transduced cells. (XLSX 114 kb)

Supplementary Table 3 List of significantly enriched canonical pathways detected by IPA core analysis. (XLSX 18 kb)

Supplementary Table 4 List of significantly enriched upstream regulators detected by IPA core analysis. (XLSX 166 kb)
