# Supplementary figures and images for: Cooperating, congenital neutropenia–associated Csf3r and Runx1 mutations activate pro-inflammatory signaling and inhibit myeloid differentiation of mouse HSPCs
Source: Ann Hematol. 2020 Aug 3;99(10):2329–38. doi: 10.1007/s00277-020-04194-0 (PMC7481169; doi:10.1007/s00277-020-04194-0)

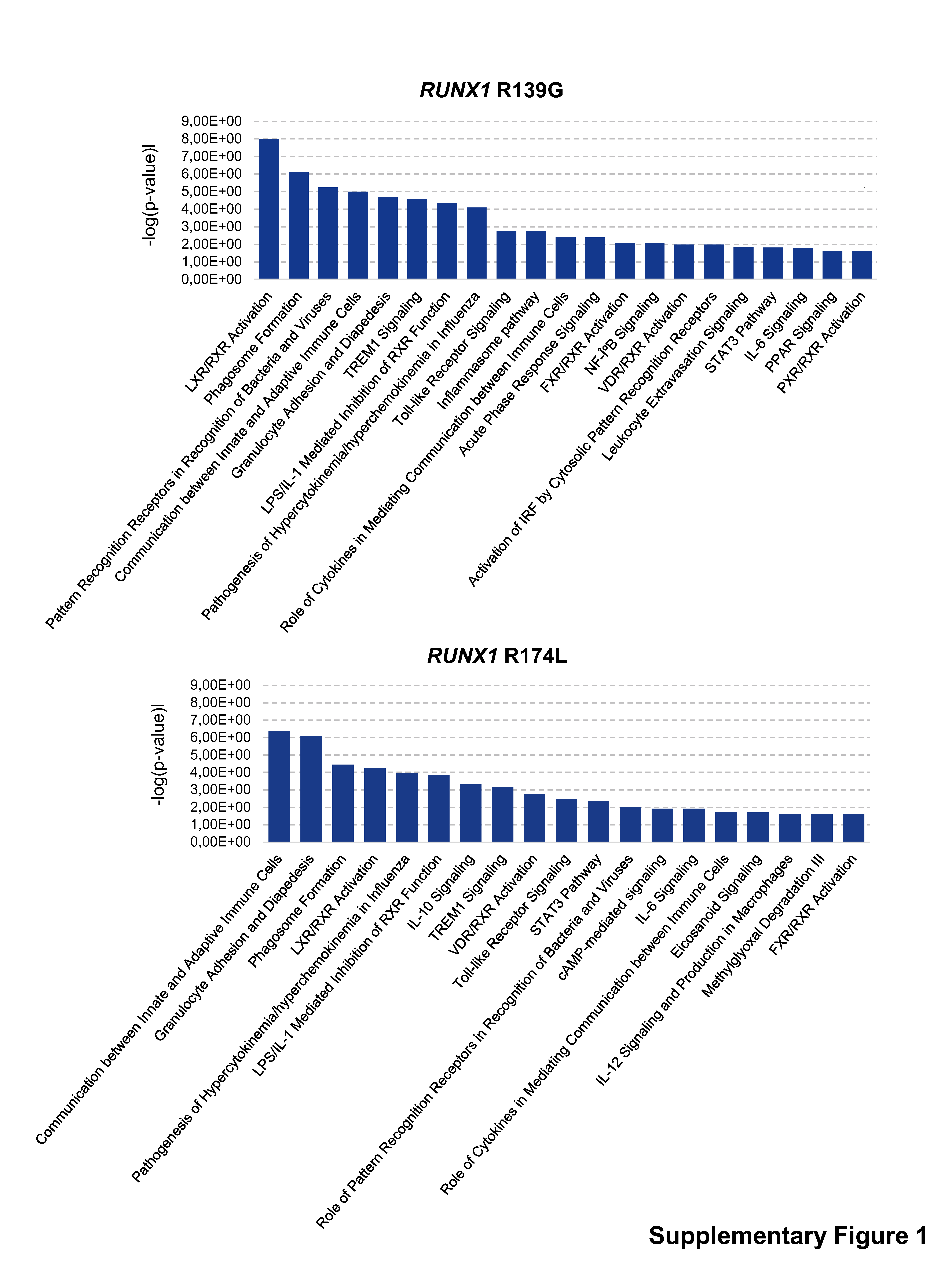

Supplement: Supplementary file 1 — Selected significantly enriched canonical pathways detected by IPA core analysis. (PNG 610 kb) [file 277_2020_4194_Fig5_ESM.png]

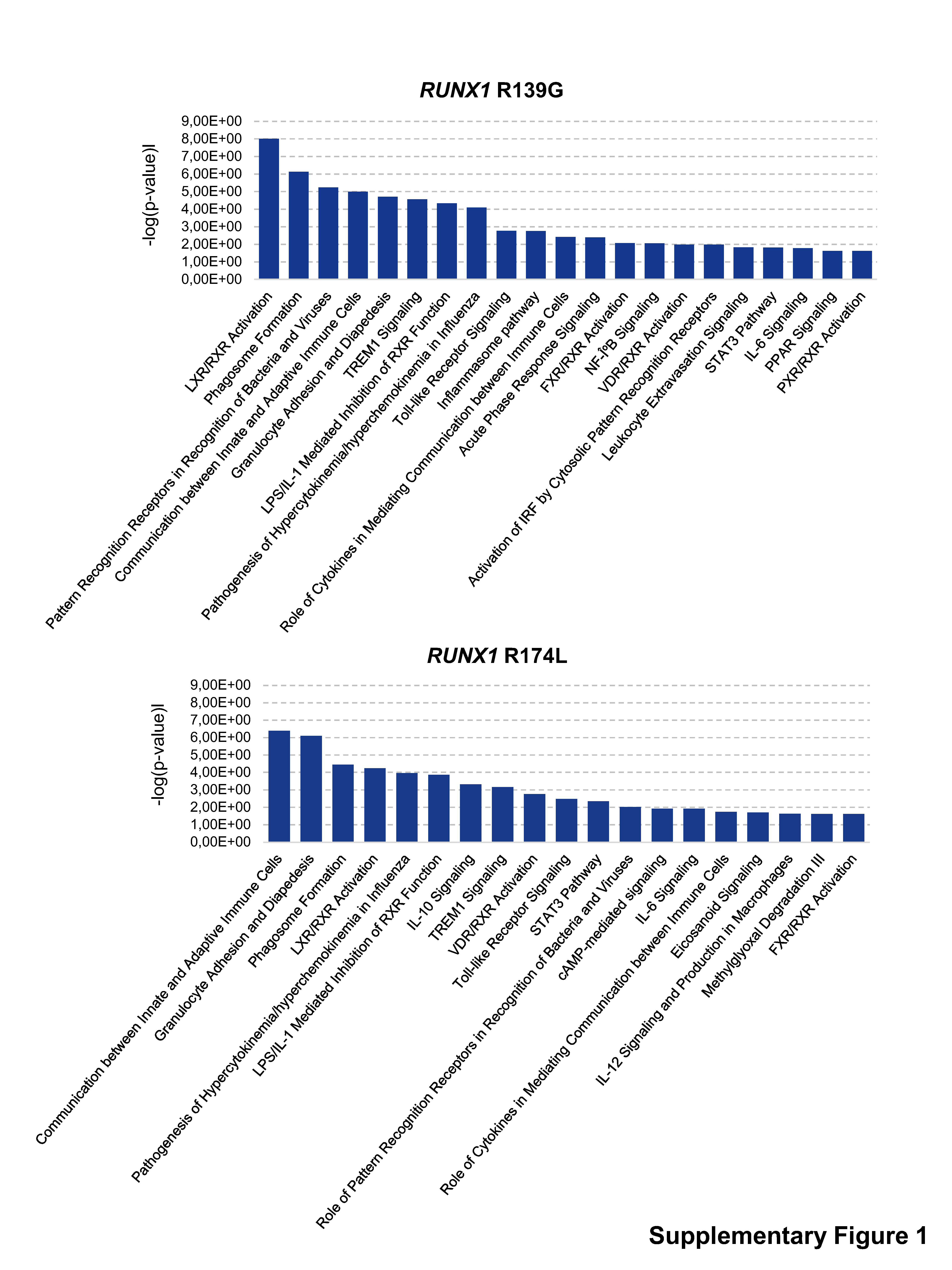

Supplement: Supplementary file 2 — High resolution image (TIF 1568 kb) [file 277_2020_4194_MOESM1_ESM.tif]

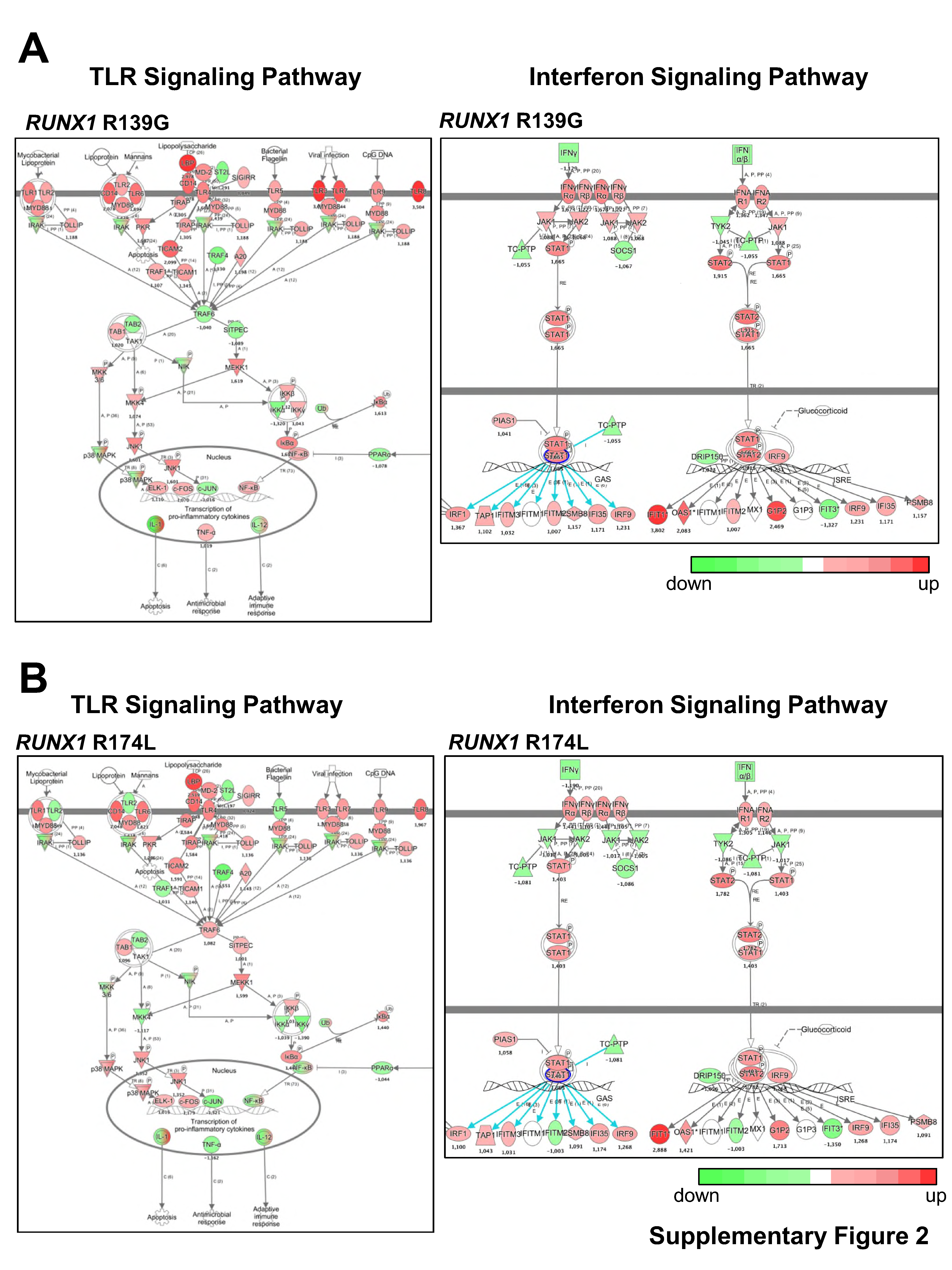

Supplement: Supplementary file 3 — TLR and IFN pathways are hyper-activated in d715 Csf3r HSPCs transduced with RUNX1 mutants, as compared to WT RUNX1 transduced cells. Pathway analysis of differentially expressed genes between WT RUNX1-overexpressing d715 Csf3r lin- cells and RUNX1-R139G- (A) or RUNX1-R174L (B) transduced cells was conducted using IPA software. Selected canonical signaling pathways (left: TLR signaling, right: Interferon signaling) that are activated in the presence of mutated RUNX1, in comparison to WT RUNX1 are depicted. The overlay of all matching molecules of selected pathways in the IPA dataset is shown. Genes marked in red are upregulated in RUNX1-mutant groups, as compared to WT-RUNX1 sampels, while green coloured genes are downregulated. (PNG 3476 kb) [file 277_2020_4194_Fig6_ESM.png]

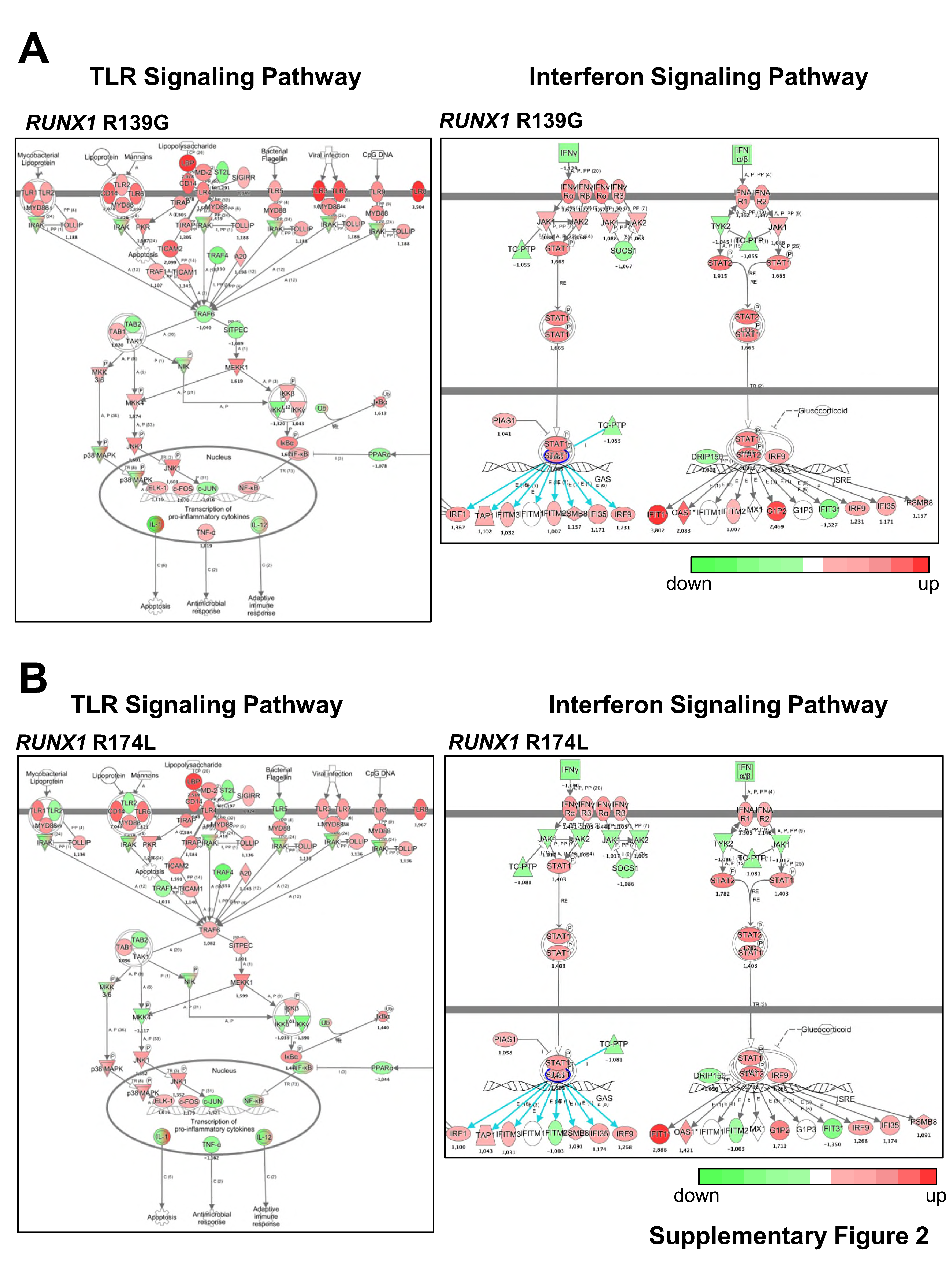

Supplement: Supplementary file 4 — High resolution image (TIF 7868 kb) [file 277_2020_4194_MOESM2_ESM.tif]

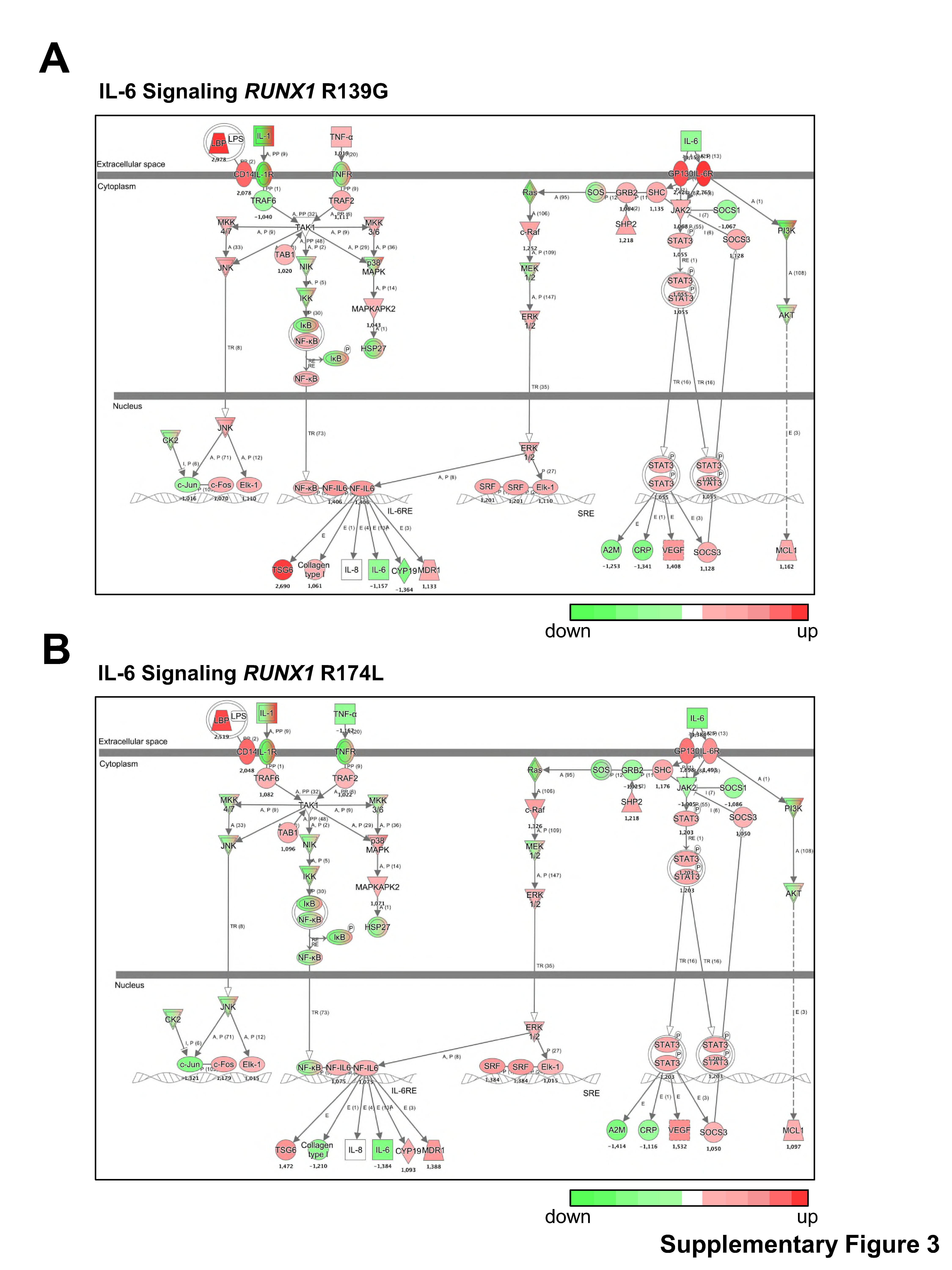

Supplement: Supplementary file 5 — IL-6 signaling is upregulated in d715 Csf3r HSPCs transduced with RUNX1 mutants. Overlays for IL-6 signaling pathway of all matching molecules contained in the IPA dataset demonstrate pathway activation in A, RUNX1-R139G and B, RUNX1-R174L overexpressing d715 Csf3r lin- cells compared to WT RUNX1 overexpressing cells. Green represents negative expression fold change while red marks positive expression fold change. Genes marked in red are upregulated in RUNX1-mutant groups, as compared to WT-RUNX1 sampels, while green coloured genes are downregulated. (PNG 2433 kb) [file 277_2020_4194_Fig7_ESM.png]

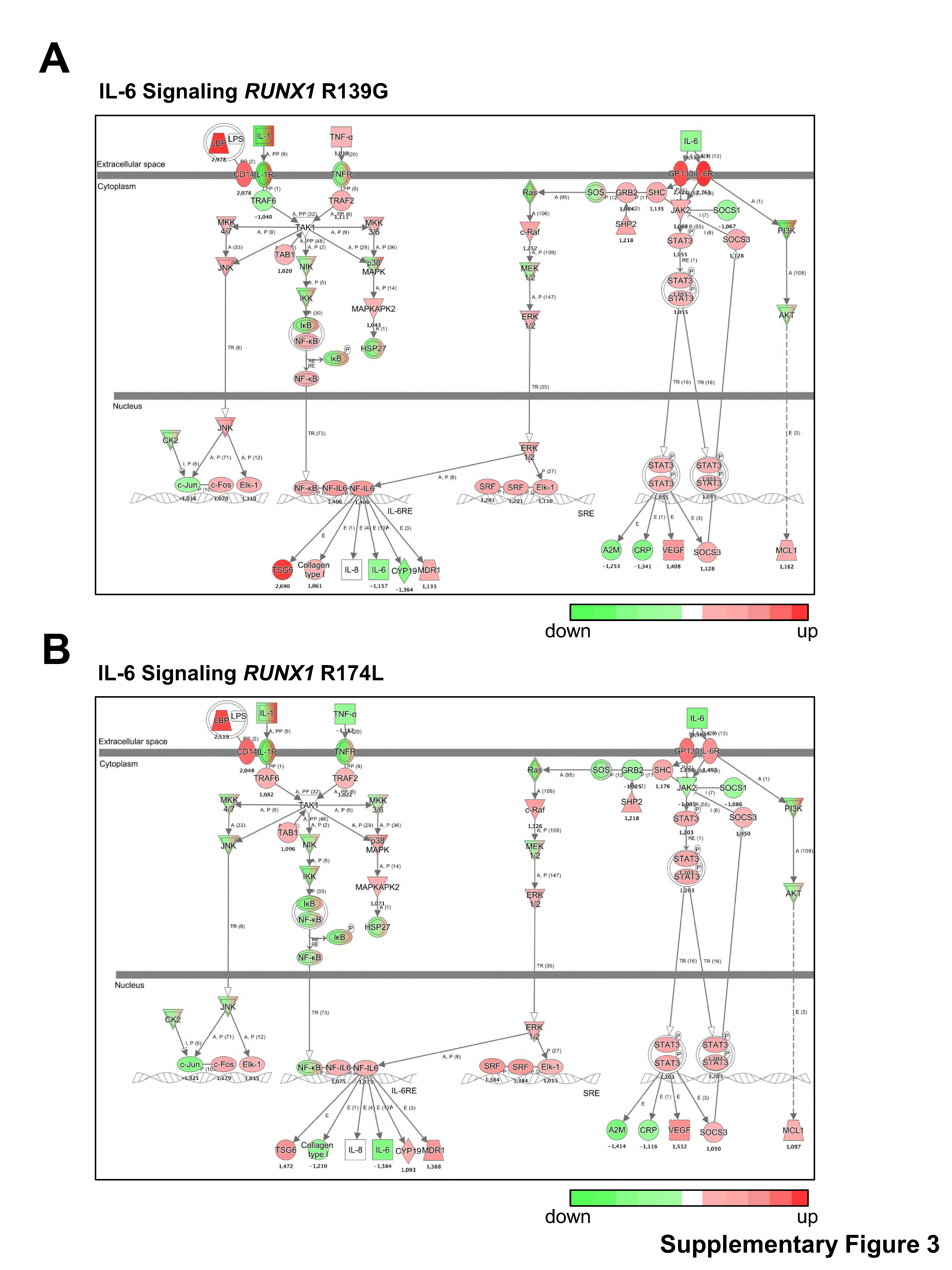

Supplement: Supplementary file 6 — High resolution image (TIF 6083 kb) [file 277_2020_4194_MOESM3_ESM.tif]

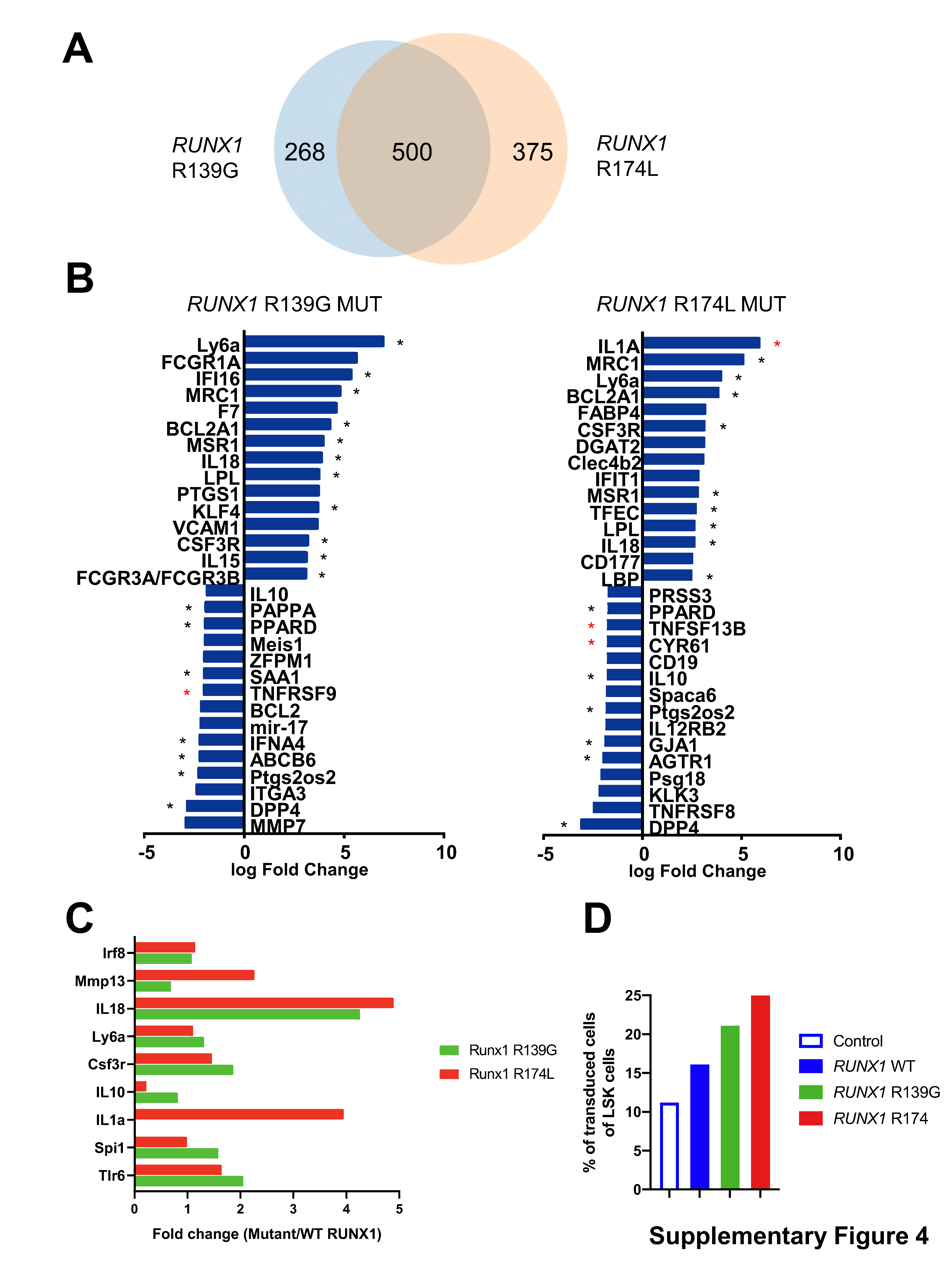

Supplement: Supplementary file 7 — Analysis of the upstream regulators responsible for the described phenotypes. A, Venn diagram depicting the overlay of all significantly enriched upstream regulators. B, Among all significantly enriched upstream regulators found by IPA core analysis, the 15 top up- and downregulated upstream regulators are shown. Black stars mark genes that are differentially expressed in the same direction in both RUNX1 mutants; red stars mark genes that are expressed oppositely. C, qRT-PCR of the selected candidate genes that were identified in the microarray analysis. The fold change of expression differences of cells transduced with RUNX1 mutants normalized to RUNX1 wildtype transduced samples is shown. D, Flow cytometry analysis of LSK cells in d715 Csf3r lin- cell population transduced with RUNX1-WT, or RUNX1 mutants and treated with G-CSF, as described in the Material and Methods section. Representative results of one donor mouse are shown. (PNG 599 kb) [file 277_2020_4194_Fig8_ESM.png]

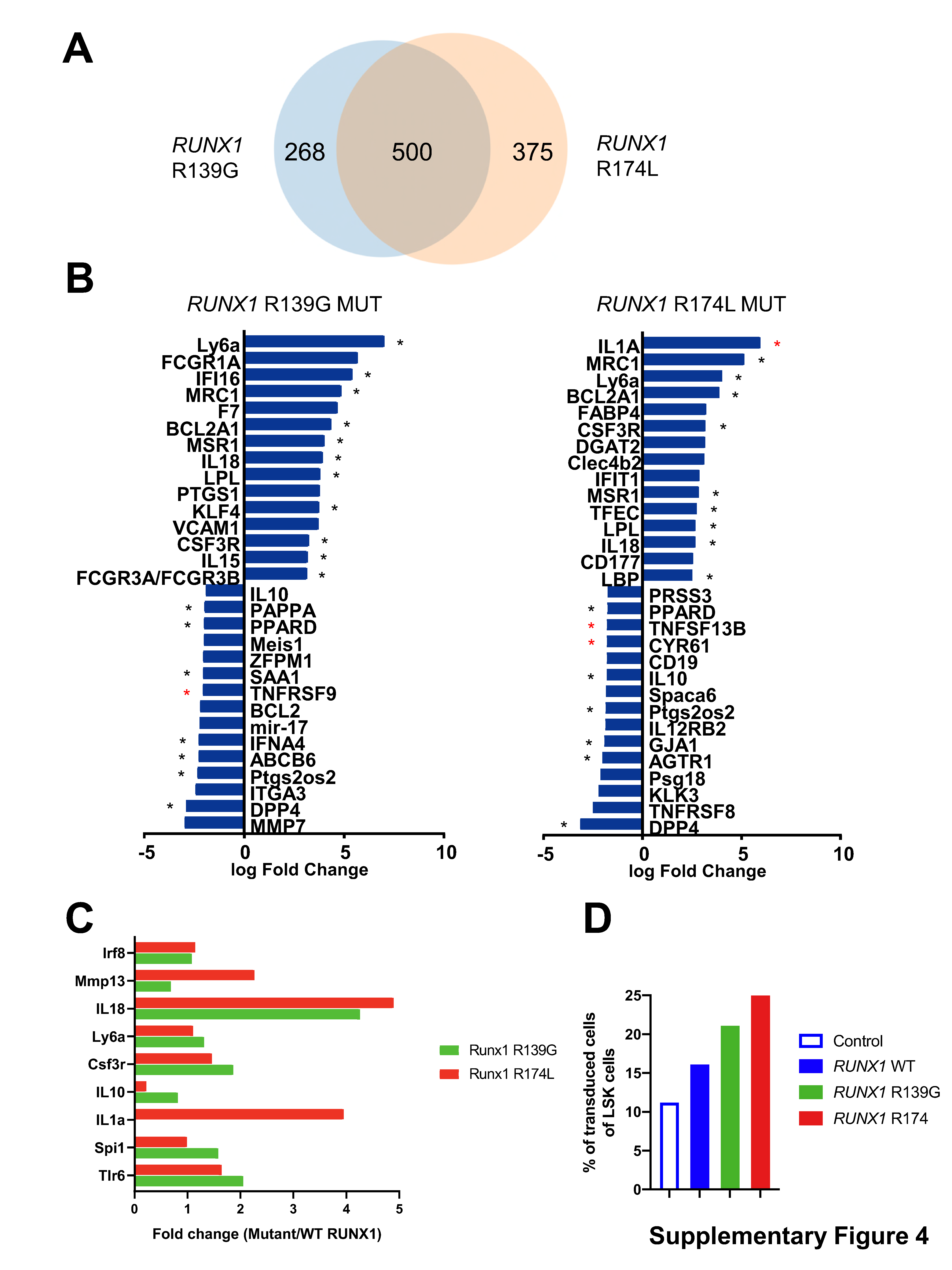

Supplement: Supplementary file 8 — High resolution image (TIF 1676 kb) [file 277_2020_4194_MOESM4_ESM.tif]

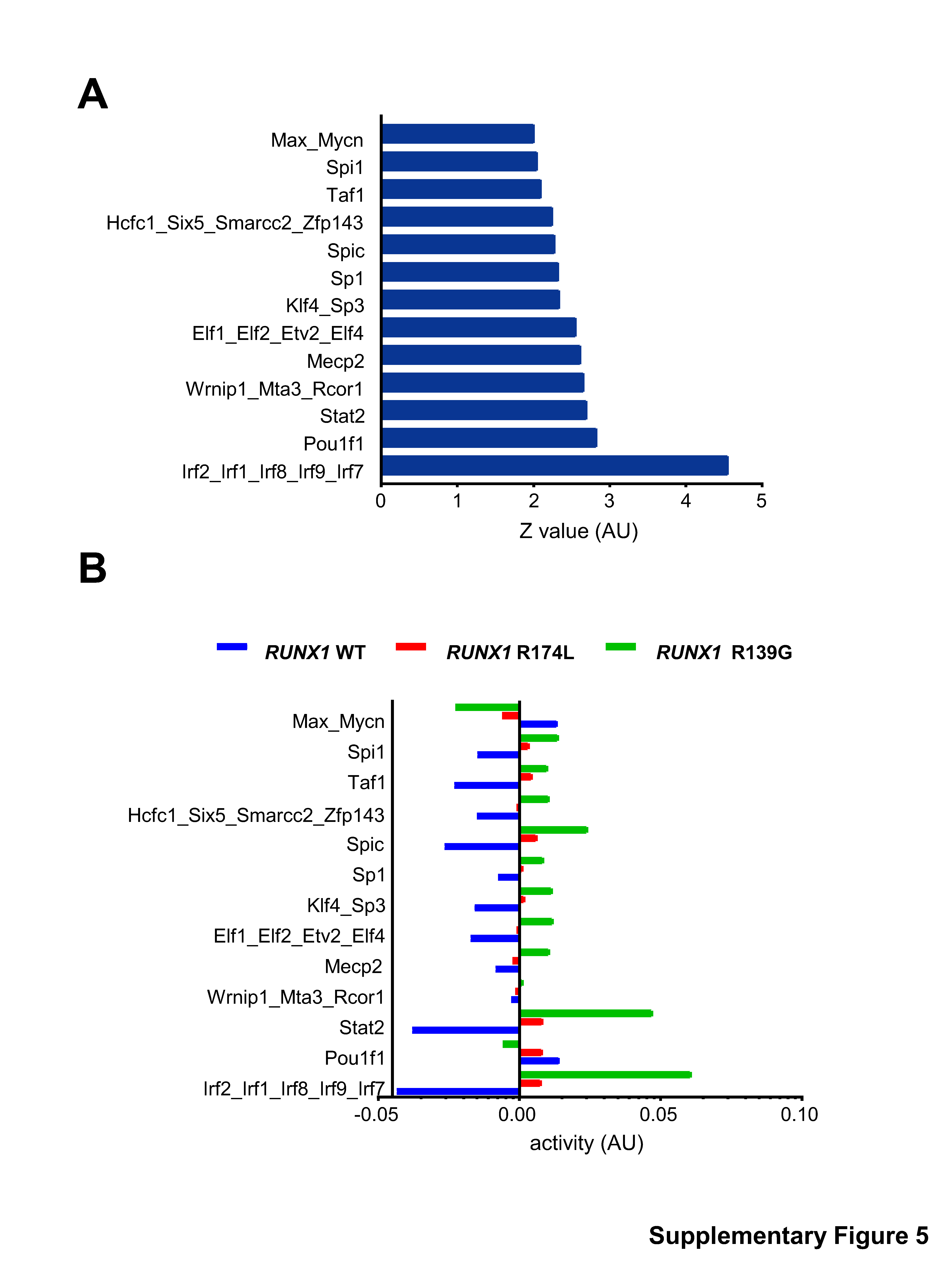

Supplement: Supplementary file 9 — Motif Activity Response Analysis performed using the ISMARA webtool for d715 Csf3r lin- cells transduced with WT RUNX1 or the RUNX1 mutants A, Significant active motifs found to be responsible for the observed differential gene expression patterns in d715 Csf3r lin- cells transduced with RUNX1 mutants compared to WT RUNX1-overexpressing cells (z-value >2). B, Activity scores (arbitrary unit) for each motif in the variously transduced cells. (PNG 253 kb) [file 277_2020_4194_Fig9_ESM.png]

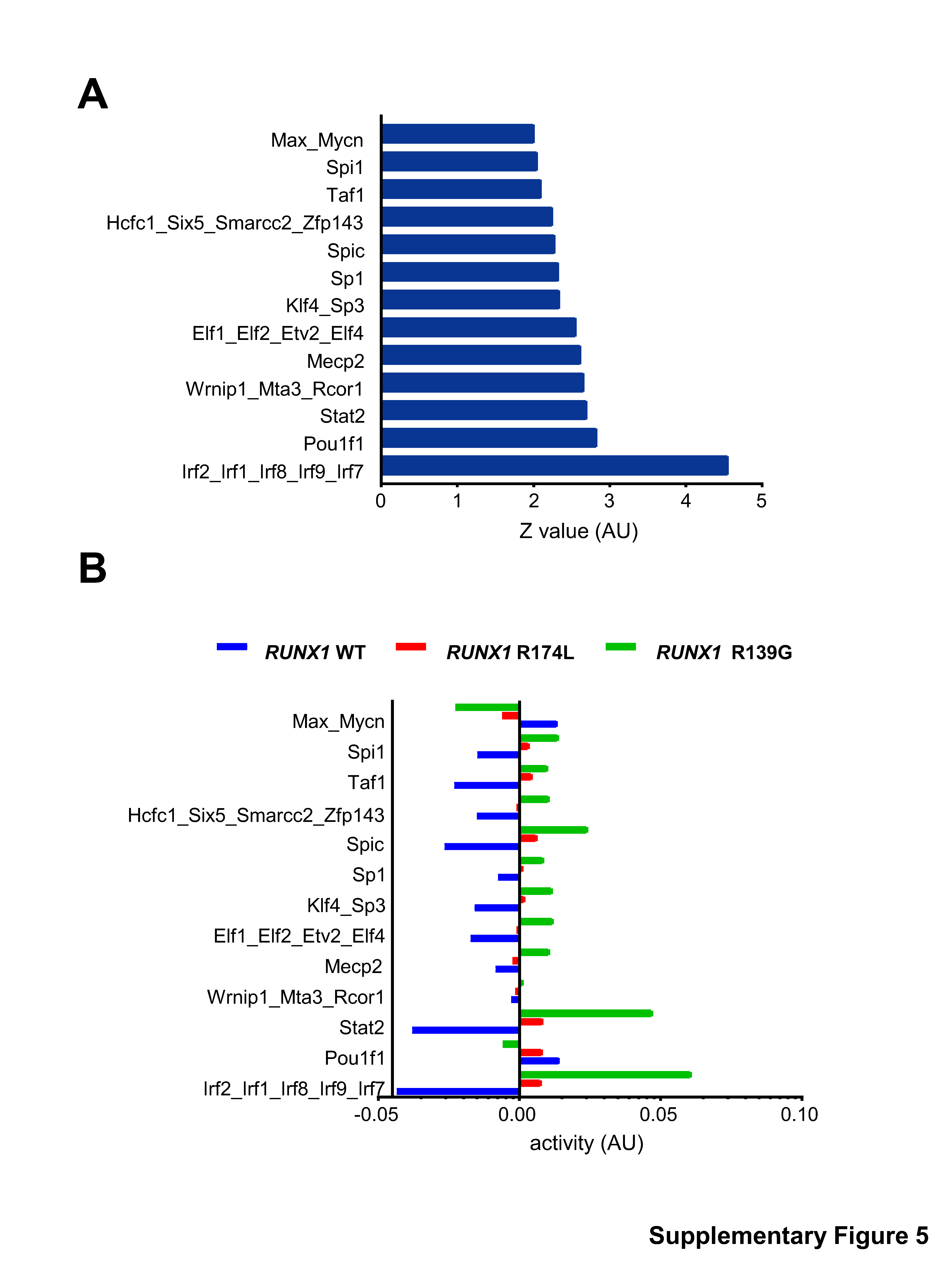

Supplement: Supplementary file 10 — High resolution image (TIF 1316 kb) [file 277_2020_4194_MOESM5_ESM.tif]
